# Acupuncture for benign prostatic hyperplasia in the elderly: A systematic review of acupoints

**DOI:** 10.1097/MD.0000000000043802

**Published:** 2025-08-08

**Authors:** Chen Guo, Lifeng An, Geling Lu, Jingwen Huang

**Affiliations:** a Graduate School, Heilongjiang University of Chinese Medicine, Harbin, China; b Department of Basic Chinese Medicine, Jiamusi Campus of Heilongjiang University of Chinese Medicine, Jiamusi, China.

**Keywords:** acupoints statistical, acupuncture, benign prostatic hyperplasia in the elderly, clustering analysis

## Abstract

**Background:**

Benign prostatic hyperplasia (BPH) affects >50% of males aged ≥50 years, causing lower urinary tract symptoms that significantly impair quality of life. While acupuncture is increasingly used for BPH management, its acupoint selection patterns remain unstandardized.

**Methods:**

Clinical studies on acupuncture/moxibustion for BPH published before September 30, 2024, were retrieved from CNKI, WANFANG, VIP, PubMed, and ScienceDirect. Acupoint patterns were analyzed using the Traditional Chinese Medicine Inheritance Assistance Platform (TCMISS).

**Results:**

Among 270 articles, 85 met the inclusion criteria. Sixty-one acupoints were identified, with high-frequency selections including Guanyuan (CV4), Zhongji (CV3), Sanyinjiao (SP6), Qihai (CV6), Shenshu (BL23), Shuidao (ST28), Pangguangshu (BL28), Yinlingquan (SP9), Qugu (CV2), and Ciliao (BL32). The Conception Vessel (CV), Bladder (BL), and Spleen (SP) meridians were predominantly used, primarily distributed in the chest/abdomen (EX-CA) and back/waist (EX-BW) regions. Association rule analysis revealed strong correlations among acupoints, with key combinations being Zhongji (CV3)-Sanyinjiao (SP6), Guanyuan (CV4)-Qihai (CV6), and Guanyuan (CV4)-Sanyinjiao (SP6). Core therapeutic clusters centered on Zhongji (CV3), Guanyuan (CV4), and Shenshu (BL23), integrated with Sanyinjiao (SP6), Shuidao (ST28), and Yinlingquan (SP9).

**Conclusions:**

Acupuncture for BPH primarily targets CV4, CV3, SP6, CV6, BL23, ST28, BL28, SP9, CV2, and BL32, reflecting their strong therapeutic relevance. These findings highlight meridians and acupoints potentially critical for symptom alleviation. However, rigorous clinical trials are warranted to validate efficacy and optimize protocols. This review provides a foundation for advancing evidence-based acupuncture interventions in BPH management.

## 1. Introduction

Benign prostatic hyperplasia (BPH) is a degenerative urinary disease with high incidence in middle-aged and elderly men worldwide.^[1–3]^ The pathological features of BPH are characterized by overactive urinary tract symptoms (lower urinary tract symptoms (LUTS)) caused by an imbalance in prostate mesenteric-epithelial cell proliferation, including bladder irritation symptoms such as frequency, urgency, and nocturia, prolonged urination time, intermittent urinary flow, and progressive dysuria.^[4,5]^ Epidemiological data show that BPH is the most common urinary disease and seriously affects the quality of life (QOL) of elderly men, with an age-standardized prevalence of 50% to 75% in men aged ≥50 years and a significant age-dependent increase (up to 82% in those aged ≥70 years).^[6,7]^ With the aging of the population, the limitations of traditional Western medical treatment (such as drugs and surgery) have gradually emerged: α-receptor blockers easily cause orthostatic hypotension, 5α-reductase inhibitors have the risk of sexual dysfunction, and minimally invasive surgery is effective; however, postoperative complications and high cost limit its popularity.^[8]^ In the era of Precision Medicine 2.0, the diagnosis and treatment of BPH are undergoing a paradigm shift from lesion removal to microenvironment remodeling.^[9,10]^ Therefore, as the “green therapy,” acupuncture has gradually become a hot research topic in international urology due to its high safety, few side effects, and overall regulatory advantages.^[11–13]^

The latest progress in evidence-based medical research shows that acupuncture has formed a multi-dimensional evidence chain in the field of BPH treatment.^[14]^ In a randomized controlled trial involving 100 patients with moderate and severe BPH, electroacupuncture significantly improved the International Prostate Symptom Score (IPSS).^[15,16]^ At week 6, the reduction in IPSS in the treatment group was 4.51 (*P* < .001) higher than that in the control group. At week 18, an increase of 3.2 points in IPSS reduction was found in the treatment group (*P* = .001), indicating that EA improved patients’ QOL, and acupoints demonstrated superior efficacy to non-acupoints in treating BPH.^[15]^ Acupuncture-moxibustion is effective and safe for treating BPH of different degrees (mild, moderate, and severe) and LUTS caused by BPH.^[17,18]^ At present, there are 3 innovative trends in the clinical practice of acupuncture and moxibustion for BPH: the innovation of acupuncture and devices (the optimization of electroacupuncture parameters, the warming effect of fire needling, and the penetrating needling technique of elongated needles), improved needling techniques (a study on the effect difference between deep needling (≥75 mm) and shallow needling (≤25 mm) at the sacral acupoints of Ciliao (BL32) and Zhongliao (BL33), and combined therapy (acupuncture, moxibustion, and acupoint injection).^[16,17,19–21]^ Among these, electroacupuncture therapy provides space for the optimization of acupuncture parameters in clinical applications. Deep needling (approximately 60–75 mm) and shallow needling (approximately 25–40 mm) BL 32 and BL 33 can improve LUTS. Deep EA at BL 32 and BL 33 can significantly enhance the scores of international prostate symptoms (IPSS decreased ≥10.51 points) and QOL in patients with BPH.^[16]^ It is worth noting that current research on the acupoint compatibility rules of acupuncture and moxibustion in treating BPH has obvious limitations. Although “Guanyuan” (CV 4), “Zhongji” (CV 3), and “Sanyinjiao” (SP 6) have been widely established as the core acupoints, the acupoint matching schemes of different academic schools and regional characteristics are significantly heterogeneous in clinical practice. What is more outstanding is that the existing studies are mostly limited to the biological mechanism of the single acupoint effect and lack a systematic study on the dynamic association model of “acupoint-meridian-zangfu,” and generally ignore the individualized regulation needs of patients’ constitution classification and disease course stage. Based on this, this study integrated the prescription mining, complex network analysis, and data visualization modules of the TCM inheritance computing platform to analyze the acupoint selection rules of BPH acupuncture and moxibustion treatment through multi-dimensional analysis (frequency statistics → association rules → entropy clustering), focusing on screening high-frequency acupoint combinations with a synergistic effect. This study provides data support for precise and individualized acupoint prescription optimization in clinical practice, which has important practical value in improving the stability of therapeutic effects and reducing medical costs.

## 2. Data and methods

### 2.1. Sources of information

A comprehensive literature search was conducted without restrictions on language. Systematic searches were performed across Chinese databases (CNKI, WANFANGDATA, and VIP) and international databases (PubMed, ScienceDirect), supplemented by other relevant sources. For Chinese databases, the search strategy utilized the Boolean query (“前列腺增生” OR “BPH”) AND (“针灸” OR “针刺” OR “艾灸”), restricted to clinical studies published before September 30, 2024. For English databases, the strategy employed (“prostatic hyperplasia” OR BPH) AND (acupuncture OR acupoint* OR moxibustion), with results filtered to include clinical trials published before September 30, 2024.

No relevant studies were identified in PubMed and ScienceDirect. This finding may be attributable to several factors: First, a significant language bias exists, as the majority of clinical trials investigating acupuncture for BPH are predominantly published in Chinese-language journals. Second, limitations in the search strategy, particularly the reliance on English keywords, may have failed to capture studies employing alternative terminology (e.g., “prostate hyperplasia” without the “benign” qualifier) or region-specific nomenclature for acupoints. Third, there appears to be limited availability of clinical trials conducted in other country settings specifically examining BPH-targeted acupoint combinations. In addition, the disciplinary focus within the English urological literature tends to prioritize pharmacological and surgical treatment over complementary and alternative therapies, such as acupuncture. Collectively, these factors underscore the critical importance of integrating cross-lingual and cross-cultural research approaches in future investigations of acupuncture for BPH.

### 2.2. Inclusion criteria

1. Patients with a clear diagnosis of BPH regardless of race, condition, or intensity;
2. clinical reports on the use of acupuncture in the treatment of BPH;
3. clear syndrome differentiation and acupoint prescription;
4. The types of interventions that included combination treatments, such as tuina therapy, physiotherapy, and placebo, were retained.

### 2.3. Exclusion criteria

1. The treatment methods were eye acupuncture, ear acupuncture, 7-star needle acupuncture, acupotomology, warm needling therapy, blade acupuncture, or balance acupuncture.
2. News reports and lectures;
3. Articles that cannot be identified only by the abstract and the full text are difficult to obtain.
4. EndNote software was used to eliminate duplicate studies, and only 1 article was used for data extraction.

### 2.4. Literature screening

The searched literature was independently screened according to the inclusion and exclusion criteria, the extracted data were cross-checked, and disagreements were resolved through internal discussion. The title and abstract were read first, after which the full text was read to determine whether they could be included in the study. The names of the acupoints were standardized. According to the data specification, the “National Standard Acupoint Names and Location of the People’s Republic of China,” the Nomenclature, and the location of meridian points.

### 2.5. Data processing

The selected data were used to establish an acupoint prescription database imported into the Chinese Medicine inheritance auxiliary platform for descriptive and association rule analysis. Association rules can uncover the mutual influence and causal structure among item sets within a large amount of data. An item set pattern that meets and exceeds the degree of support is regarded as a frequent item set. Through the analysis of frequent item sets, high-frequency acupoint combinations in acupuncture treatment prescriptions can be summarized, and the combination rules can be inferred. In the analysis of association rules, the number of support degrees represents the frequency of the simultaneous occurrence of acupoints X and Y, the support degree represents the frequency of the simultaneous presence of acupoints X and Y, and the confidence degree represents the probability of the appearance of acupoint Y when acupoint X emerges (the closer this parameter is to 1, the higher the probability of the appearance of 1 acupoint following the appearance of the other). Based on the abovementioned method, acupoints with close connections in acupuncture treatment prescriptions can be identified, namely, commonly used acupoint combinations.

### 2.6. Management of lost data

When important data is missing, attempt to contact the corresponding author.

### 2.7. Ethical considerations

This study is a systematic review of published literature and does not involve direct collection of human or animal data. According to international guidelines for systematic reviews (PRISMA-ScR), ethical approval and patient consent are not required for such studies.

## 3. Results and discussion

### 3.1. Literature search results

**Figure 1.**
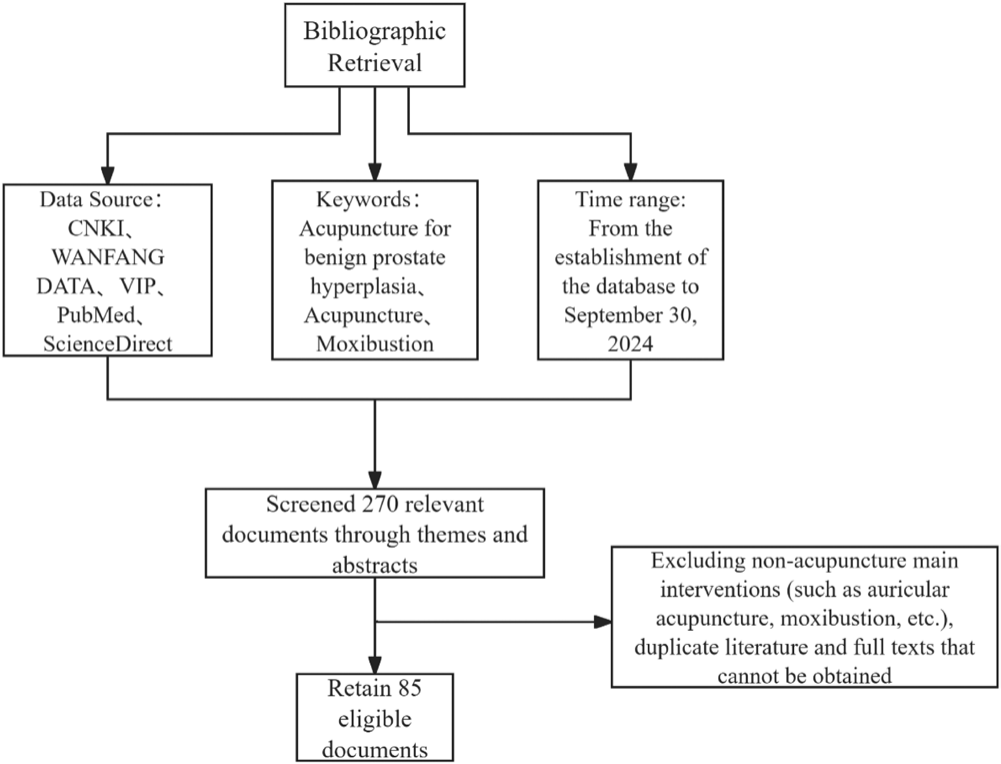
Flow chart of literature screening. A total of 270 articles were retrieved from CNKI, WANFANG, and VIP databases. After removing duplicates and applying inclusion/exclusion criteria, 85 articles were included for analysis. Boxes represent stages of screening (identification, screening, eligibility, inclusion). Arrows indicate the flow of study selection, with exclusion reasons noted (e.g., nonrelevant interventions, unavailable full texts). CNKI = China National Knowledge Infrastructure, VIP = VIP database, WANFANG = WANFANG data.

A total of 270 relevant articles were retrieved, including 195 from CNKI, 39 from Wanfang Database, and 31 from VIP Database. No relevant articles were found in PubMed and ScienceDirect. The original data downloaded were verified by 2 members (An LF and Lu GL). The flowchart of the study inclusion is shown in Figure 1. After verification, the data were imported into the Traditional Medicine Inheritance Auxiliary Platform (TCMISS) for subsequent analysis.

### 3.2. Statistical analysis of the frequency of acupoints

The TCMISS traditional Chinese medicine (TCM) inheritance auxiliary platform was used to calculate the frequency of 85 articles that met the criteria. A total of 450 occurrences of acupoints were included, with 61 unique acupoints. Acupoints were sorted according to their frequency of occurrence. Due to the excessive number of acupoints, only those used more than 9 times were included in this study, as shown in Table 1. According to the analysis of the results of acupoint selection, Guanyuan (CV4), with the most frequent acupoint selection, belongs to the conception vessel (CV), which is located in the lower abdomen, 3 cun in the middle and lower umbilicus, and the anterior midline. Guanyuan (CV4) is the alarm point of the small intestine and the crossing point between the CV and the meridians of the liver, spleen, and kidney. Detrusor dysfunction is one of the manifestations of bladder dysfunction in patients with BPH is detrusor dysfunction.^[22]^ Detrusors are not controlled or contracted in a timely and effective manner, which leads to frequent urination and endless urination.^[23]^ Zhongji (CV3) is the acupoint of the CV, which is located in the lower abdomen, 4 cun in the middle and lower umbilicus, and anterior median line. Zhongji (CV3) is the alarm point of the bladder and the crossing point between the CV and the meridians of the liver, spleen, and kidney. The inferior nerve at the Zhongji (CV3) and Guanyuan (CV4) points originates from T12-L1, which contains the pelvic nerve that innervates the bladder detrusor. Acupuncture at Zhongji (CV3) stimulates the parasympathetic nerve and inhibits the sympathetic nerve, resulting in contraction of the bladder detrusor and relaxation of the internal sphincter. It can also improve the function of the bladder detrusor, promote the timely autonomous discharge of urine, and relieve the symptoms of frequent and endless urination.^[24]^

**Table 1:** Frequency of acupoints.

Acupoint	Frequency	Proportion (Total frequency = 450) (95% CI)	Acupoint	Frequency	Proportion (Total frequency = 450) (95% CI)
CV4	59	13.1% (10.3–16.5)	SP9	17	3.8% (2.3–6.0)
CV3	52	11.6% (8.9–14.7)	CV2	17	3.8% (2.1–5.5)
SP6	33	7.3% (5.1–10.1)	BL32	16	3.6% (2.1–5.7)
CV6	31	6.9% (4.7–9.6)	BL54	14	3.1% (1.5–4.7)
BL23	29	6.4% (4.4–9.0)	KI3	12	2.7% (1.2–4.2)
ST28	23	5.1% (3.3–7.5)	LR3	9	2.0% (0.7–3.3)
BL28	19	4.2% (2.6–6.5)	ST36	9	2.0% (0.7–3.3)

Proportion = $\;\frac{Frequency}{Total\;frequency}\;$ × 100%. The 95% confidence interval was calculated using the Wilson score method.

BL = bladder meridian, CI = confidence interval, CV = conception vessel, KI = kidney meridian, LR = liver meridian, SP = spleen meridian, ST = stomach meridian.

Sanyinjiao (SP6) belongs to the spleen meridian (SP). It is located at the medial side of the calf, 3 cm above the tip of the medial malleolus, and behind the medial edge of the tibia. This is the crossing point of the liver, spleen, and kidneys. Qihai (CV6) is an acupoint of the CV, which is located in the lower abdomen, 1.5 cun below the umbilicus and on the anterior midline. Shenshu (BL23) is a point on the bladder meridian (BL), located in the lumbar region of the back, specifically 1.5 cun lateral to the posterior midline, beneath the spinous process of the second lumbar vertebra (L2). The high-frequency selection of CV3 and CV4 reflects their neuroanatomical relevance: These acupoints overlie the T12–L1 spinal segments that innervate the bladder detrusor muscle. Stimulation here enhances parasympathetic activity, promoting detrusor contraction and sphincter relaxation – directly addressing BPH-related urinary retention.^[24]^ Similarly, BL23 correlates with sacral micturition centers (S2–S4), regulating bladder storage/voiding reflexes.^[25]^

### 3.3. Statistical analysis of meridian frequency

**Figure 2.**
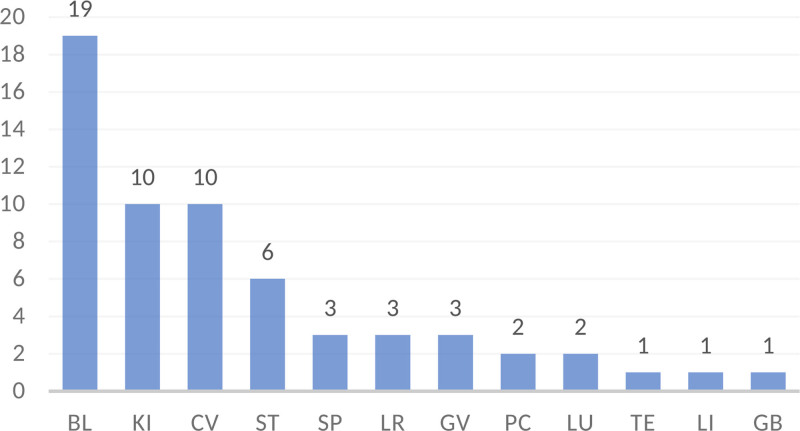
Bar chart of the frequency of meridian application. The CV, BL, and SP were the most frequently used meridians, with frequencies of 173, 120, and 55, respectively. Y-axis: frequency; X-axis: meridians. Error bars represent 95% confidence intervals (Wilson Score method). BL = bladder meridian, CV = conception vessel, SP = spleen meridian.

According to the statistical results of the frequency of acupoints, the meridians and acupoints were classified into 12 meridians. As shown in Figure 2, the most frequently involved meridians were the CV (173), bladder meridian (BL) (120), and spleen meridian (SP) (55). The lung meridian (LU), pericardium meridian (PC), triple energizer meridian (TE), large intestine meridian (LI), and gallbladder meridian (GB) were not more than 2 times.

The most frequently treated acupoints were Guanyuan (CV4) at the CV, Zhongji (CV3), and Sanyinjiao (SP6) at the spleen meridian (SP), with 59, 52, and 33 times, respectively, as shown in Table 2. Guanyuan (CV4) and Zhongji (CV3) are the most commonly used acupoints in the CVs. CV, which was consistent with the results of a previous statistical analysis of the acupoint frequency. These results indicate that CV acupoints are more frequently used in the treatment of BPH in the elderly. The effect of acupuncture on the regulation of urination function is closely related to the local neuroanatomical structure of the selected acupoints. The peripheral nervous system that controls the bladder and urethra mainly includes the pelvic, lower abdominal, and perineal nerves.^[25–27]^ Studies have found that the nerves near the acupuncture points that affect urethral function project to the spinal cord segments from L1 to S5, which is highly consistent with the segment of the urethral nerve entering the spinal cord.^[28]^ The CV was first recorded in Huangdi Neijing (Yellow Emperor’s Internal Canon), the sea of Yin vessels, including any Yin vessels in the body. The CV runs from the lower abdomen, down the perineum, and upward through the pubis. TCM theories suggest that BPH is related to the imbalance of Yin and Yang in the kidney, spleen, and other viscera. By regulating the CV qi and blood, Yin of the human body can be regulated to improve the local qi and blood state of the prostate. If the qi and blood of the CV are not smooth, this may lead to qi and blood stasis in the lower jiao, including the pelvic part where the prostate is located. Acupuncture at the points of the CV can play a role in clearing qi and blood, improving the blood circulation of the prostate, and reducing the compression of the surrounding tissues, such as the urethra, byhyperplasia tissue.^[29]^ The bladder meridian (BL) is located outside and inside the bladder bowels. Patients with BPH often have voiding dysfunction, such as frequent urination, urgency, and incontinence, and the relaxation function of the bladder is closely related to these voiding symptoms. The gasification function of the bladder can be regulated by acupuncture at the bladder meridian (BL). The patency of qi and blood at the bladder meridian (BL) also helps reduce local qi and blood stasis and relieve discomfort symptoms such as pain caused by prostatic hyperplasia. Acupuncture at the spleen meridian (SP) can invigorate the spleen and reduce dampness, help to improve the metabolism of water and fluid in the body, reduce local edema and other conditions that may be accompanied by prostatic hyperplasia, and improve urination and other related symptoms. The stomach meridian (ST) has a wide circulation range in the human body, and its qi and blood circulation can affect multiple body parts. Although the stomach meridian does not pass directly through the prostate, it is closely related to the circulation of qi and blood in the abdomen. According to the principle of “where the meridians pass, the indications are,” acupuncture at the points of the stomach meridian can regulate the overall circulation of qi and blood in the abdomen and improve the qi and blood state in the pelvis, where the prostate is located. Kidney Yang is one of the fundamentals of Yang qi in the human body, and the governing vessel is closely related to the kidney. In terms of physiological function, the warming effect of kidney yang is essential for maintaining the normal function of the urinary system, including the prostate. When the kidney yang is insufficient, urinary system diseases, such as prostatic hyperplasia, may occur. Acupuncture at the governor vessel point can stimulate Yang qi and promote circulation and distribution. Sufficient Yang qi can warm the lower jiao, improve the local blood circulation of the prostate, and reduce pathological states such as cold coagulation and blood stasis caused by prostatic hyperplasia, thereby improving symptoms such as poor urination caused by prostatic hyperplasia. In addition, the liver meridian (LR) and gallbladder meridian (GB) encircle the perineum, whereas the prostate, a part of the male reproductive system, is located in the pelvic cavity. According to TCM principles, acupoints along the liver (LR) and gallbladder (GB) meridians exhibit topographical correspondence with the prostate region. Stimulation of these acupoints is theorized to modulate local neurovascular activity, enhance pelvic circulation, and reduce prostatic tension. This neuromodulatory effect may alleviate discomfort associated with BPH and contribute to functional improvements in urinary symptoms. The dominance of the CV aligns with its trajectory through the prostate region. CV acupoints resolve Qi stagnation and blood stasis in the lower jiao – a core TCM pathogenesis in BPH.^[29]^ Concurrently, spleen meridian points like SP6 eliminate “dampness accumulation” (key etiology for LUTS), synergizing with CV’s local action through the triple-Yin confluence.

**Table 2:** Frequency of meridians.

Meridian	Acupoint	Frequency	Meridian	Acupoint	Frequency
BL (121)	BL23	29	ST (42)	ST28	23
	BL28	19		ST36	9
	BL32	16		ST29	6
	BL54	14		ST25	2
	BL33	8		ST40	1
	BL35	6		ST30	1
	BL22	6	KI (30)	KI3	12
	BL18	4		KI11	5
	BL31	3		KI12	4
	BL34	3		KI1	2
	BL17	3		KI10	2
	BL20	3		KI2	1
	BL13	1		KI8	1
	BL15	1		KI6	1
	BL57	1		KI7	1
	BL40	1		KI13	1
	BL25	1	SP (55)	SP6	33
	BL27	1		SP9	17
	BL26	1		SP10	5
CV (173)	CV4	59	LR (12)	LR3	9
	CV3	52		LR2	2
	CV6	31		LR5	1
	CV2	17	LU (4)	LU7	3
	CV8	5		LU5	1
	CV12	4	GV (27)	GV4	3
	CV10	2		GV20	2
	CV9	1		GV3	1
	CV5	1	GB (1)	GB43	1
	CV1	1	LI (1)	LI4	1
PC (3)	PC6	2	TE (2)	TE6	2
	PC7	1			

BL = bladder meridian, CV = conception vessel, GB = gallbladder meridian, GV = governor vessel, KI = kidney meridian, LI = large intestine meridian, LR = liver meridian, LU = lung meridian, PC = pericardium meridian, SP = spleen meridian, ST = stomach meridian, TE = triple energizer meridian.

Based on the above analysis, the CV and bladder meridian (BL) are central in treating BPH and show significant clinical application value.

### 3.4. Statistical analysis of the acupoints distribution in human body parts

Acupoint distribution was categorized into the following regions: head and neck (EX-HN), back and waist (EX-BW), upper extremities (EX-UE), lower extremities (EX-LE), and chest and abdomen (EX-CA). The distribution patterns are presented in Table 3. The results showed that the total number of acupoints selected was 450. The most frequently chosen acupoints were located at the chest and abdomen (EX-CA) 215 times (47.8% of the total frequency), and the total number of acupoints was 17, which is consistent with the principle of proximal acupoint selection. Acupoints were selected from the chest and abdomen. The abdominal acupoints (EX-CA) were more frequent in Guanyuan (CV4) (59), Zhongji (CV3) (52), Qihai (CV6) (31), Shuidao (ST28) (23), and Qugu (CV2) (17). The second most frequently selected acupoint was back and waist (EX-BW), 123 times in total, accounting for 27.3% of the total frequency, and the total number of acupoints selected was 19. Regarding acupoint selection of points of the back and waist (EX-BW), the principle of acupoint selection based on syndrome differentiation, symptomatic acupoint selection, and distal acupoint selection in the treatment of prostatic hyperplasia was met. The most frequently used acupoints on the back and waist (EX-BW) were Shenshu (BL23) (29), Pangguangshu (BL28) (19), Ciliao (BL32) (16), and Zhibian (BL54) (14). The acupoints of the chest and abdomen (EX-CA) and of the chest and abdomen (EX-CA) embody the treatment principle of combining proximal and distal acupoints, syndrome differentiation, and symptomatic acupoint selection. The frequency of acupoint selection at the points of the head and neck (EX-HN) was the least, 2 times, accounting for 0.4% of the total frequency. According to the results of the analysis of the acupoints mentioned above, the most frequently used acupoints were also distributed in the chest and abdomen (EX-CA) and back and waist (EX-BW). This proves that the points of the chest and abdomen (EX-CA) and back and waist (EX-BW) play a key role in treating BPH in the elderly, showing their significant potential as research objects.

**Table 3:** Distribution of acupoints by body region.

Body region	Frequency	Proportion (95% CI)
EX-CA	215	**47.8%** (43.1–52.5)
EX-BW	123	**27.3%** (23.3–31.7)
EX-LE	100	**22.2%** (18.5–26.3)
EX-UE	10	**2.2%** (1.1–4.0)
EX-HN	2	**0.4%** (0.1–1.5)

Proportion = $\;\frac{Frequency}{Total\;frequency}\;$ × 100%. The 95% confidence interval was calculated using the Wilson Score method. The bold values highlight the 2 most frequently selected body regions for acupoints in treating benign prostatic hyperplasia (BPH) in the elderly: the EX-CA with a frequency of 215 (47.8%) and the EX-BW with 123 (27.3%). This emphasis underscores their clinical significance, as these regions align with the traditional Chinese medicine principle of “proximal acupoint selection” for lower urinary tract disorders, directly targeting the anatomical and functional areas related to the prostate and bladder. Their high frequency reflects their core role in regulating qi and blood in the lower jiao, supporting the therapeutic focus on these regions in acupuncture protocols for BPH.

CI = confidence interval, EX-BW = back/waist, EX-CA = chest/abdomen, EX-HN = head/neck, EX-LE = lower extremities, EX-UE = upper extremities.

### 3.5. Frequency statistics of specific points usage

In addition to the indications of general acupoints, specific points also have functions of stronger pertinence, closer connection with zang-fu organs, more effectiveness in particular diseases and symptoms, and a wider regulation range. In clinical practice, specific points are often used to treat prostatic hyperplasia. According to the nature of the particular points, acupoints are divided into 5 transport points: source, crossing, and acupoints. Connecting point, cleft point, transport point, alarm point, confluence points of the 8 vessels, and lower sea points of the prostate, 6 bowels, and 8 meeting points.

**Figure 3.**
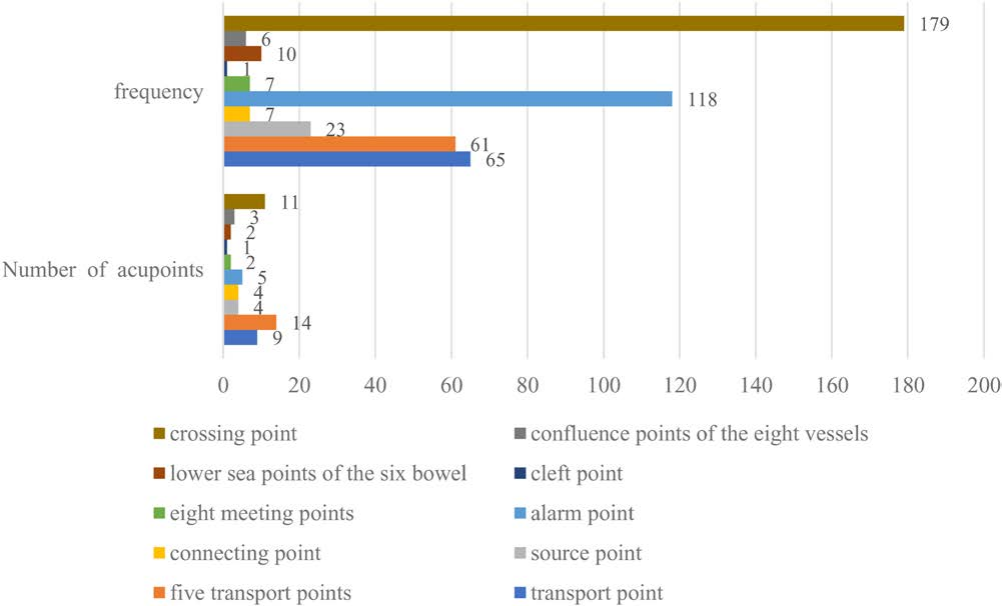
Analysis of the usage of specific acupoints. Bar chart showing the frequency of specific acupoint categories (crossing points, alarm points, 5 transport points, etc) in BPH treatment. The crossing point category (179 occurrences) was most prevalent. Colors denote different acupoint types (e.g., blue for crossing points, red for alarm points). BPH = benign prostatic hyperplasia, CV = conception vessel.

Data analysis was performed on the literature that met the standards, with a total of 55 Specific Points − 477 times. The usage frequency of each point is drawn into a bar chart, as shown in Figure 3. In BPH treatment, the crossing point was the most frequently used acupoint (179), including 11 acupoints. The most frequently used acupoints were Guanyuan (CV4), Zhongji (CV3), Sanyinjiao (SP6), and Qugu (CV2), as shown in Table 4. The results showed that Guanyuan (CV4) had the highest frequency, and Guanyuan (CV4) also belonged to the alarm point (118) with high frequency. Therefore, Guanyuan (CV4) can achieve a very good curative effect in the treatment of PH. As the most frequent crossing point, CV4 integrates liver/spleen/kidney meridians. This allows simultaneous regulation of multiple zang-fu systems involved in BPH: liver Qi stagnation (voiding difficulty), spleen dampness (urinary turbidity), and kidney deficiency (nocturia).^[29]^

**Table 4:** Frequency of use of specific points.

Specific Point	Acupoint	Frequency	Specific Point	Acupoint	Frequency
Five transport points (61)	PC7	1	Confluence points of the 8 vessels (6)	PC6	2
	ST36	9		KI6	1
	KI3	12		LU7	3
	KI1	2	Eight meeting points (7)	CV12	4
	KI10	2		BL17	3
	KI2	1	Source point (23)	PC7	1
	KI7	1		KI3	12
	TE6	2		LR3	9
	SP9	17		LI4	1
	LR3	9	Alarm point (118)	ST25	2
	LR2	2		CV4	59
	LU5	1		CV3	52
	GB43	1		CV12	4
	BL40	1		CV5	1
Lower sea points of the 6 bowel (10)	ST36	9	Connecting point (7)	PC6	2
	BL40	1		ST40	1
Crossing point (179)	ST30	1		LR5	1
	KI11	5		LU7	3
	KI12	4	Transport point (65)	BL23	29
	KI13	1		BL28	19
	CV4	59		BL22	6
	CV3	52		BL18	4
	CV2	17		BL20	3
	CV12	4		BL13	1
	CV10	2		BL15	1
	CV1	1		BL25	1
	SP6	33		BL27	1
Cleft point (1)	KI8	1			

BL = bladder meridian, CV = conception vessel, GB = gallbladder meridian, KI = kidney meridian, LI = large intestine meridian, LR = liver meridian, LU = lung meridian, PC = pericardium meridian, SP = spleen meridian, ST = stomach meridian, TE = triple energizer meridian.

### 3.6. Frequency statistics of acupoint combinations

Among the 85 articles that met the criteria, only 2 used a single acupoint. A combination of multiple acupoints usually involves acupuncture and moxibustion. To explore the relationship between acupuncture and moxibustion, a multi-acupoint combination was performed using the TCM inheritance assistance platform. The support degree was set at 12, the confidence degree was ≥0.6, and 185 acupoint combinations of acupuncture for BPH were obtained. The most frequently used combinations are Zhongji (CV3) and Sanyinjiao (SP6). Guanyuan (CV4), Qihai (CV6); “Guanyuan (CV4), Sanyinjiao (SP6),” with the frequency of 40, 27, 26, and 24, respectively, as shown in Table 5. CV3 + SP6: CV3 (local alarm point) clears lower jiao stasis, while SP6 (distal point) resolves dampness systemically. This embodies the TCM strategy “treat local symptoms + resolve root cause.” CV4 + CV6: Dual CV points synergistically tonify original Qi to improve bladder contractility in elderly BPH with kidney deficiency.^[21]^

**Table 5:** Frequency of acupoint combination.

Combination of acupoints	Frequency	Combination of acupoints	Frequency	Combination of acupoints	Frequency
CV4, CV3	40	CV3, CV6	20	CV4, CV3, CV6	16
CV3, SP6	27	CV4, ST28	20	BL23, BL28	16
CV4, CV6	26	CV4, CV3, SP6	20	CV4, CV3, ST28	16
CV4, SP6	24	CV3, ST28	19	CV4, BL28	15
CV3, BL23	23	CV4, CV3, BL23	19	CV4, SP9	15
CV4, BL23	22	SP6, BL23	17	CV3, SP6, BL23	15

BL = bladder meridian, CV = conception vessel, SP = spleen meridian, ST = stomach meridian.

### 3.7. Analysis of association rules of acupoint combination

The “association rule” analysis was performed based on the above frequency analysis. With the same support level of 12 and confidence level of ≥0.6, 16 sets of results were obtained. According to the degree of confidence, Zhongji (CV3), Guanyuan (CV4), and Shenshu (BL23) were the core acupoints. When Sanyinjiao (SP6) and Shuidao (ST28) are selected, Zhongji (CV3) should be compatible (confidence 1). Zhongji (CV3) and Pangguangshu (BL28) are often used in combination with Guanyuan (CV4) (confidence 0.92); Zhongji (CV3), Shenshu (BL23), and Pangguangshu (BL28) are often used with Guanyuan (CV4) (confidence 0.92); Sanyinjiao (SP6) and Yinlingquan (SP9) are often used with Guanyuan (CV4) (confidence 0.92); Taixi (KI3) is often used with Zhongji (CV3) (confidence 0.92); Guanyuan (CV4), Sanyinjiao (SP6), and Shenshu (BL23) are often used in combination with Zhongji (CV3) (confidence 0.92), as shown in Table 6.

**Table 6:** Association rules of acupoint combination.

Associated acupoints A	Associated acupoints B	1-a	Associated acupoints A	Associated acupoints B	1-a
SP6, ST28	CV3	1	CV3, SP9	CV4	0.91
CV3, BL28	CV4	0.92	SP9	CV4	0.88
CV3, BL23, BL28	CV4	0.92	SP6, BL23	CV3	0.88
SP6, SP9	CV4	0.92	ST28	CV4	0.87
KI3	CV3	0.92	CV4, BL28	BL23	0.87
CV4, SP6, BL23	CV3	0.92	BL54	CV3	0.86
CV4, CV3, BL28	BL23	0.92	CV4, BL23	CV3	0.86
CV3, BL28	BL23	0.92	CV4, BL23, BL28	CV3	0.85

BL = bladder meridian, CV = conception vessel, KI = kidney meridian, SP = spleen meridian, ST = stomach meridian.

### 3.8. Network topology analysis of acupoint selection

**Figure 4.**
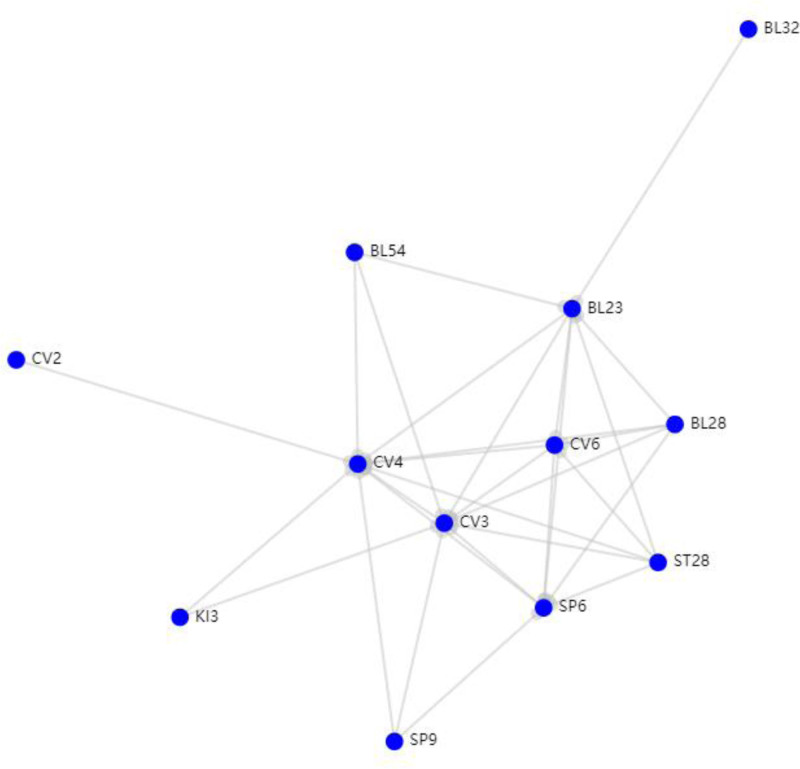
Demonstration of the acupoint combination network. Nodes represent acupoints (labeled by code, e.g., CV3, SP6), and edges indicate strong associations (support ≥12, confidence ≥0.6). Node size reflects frequency of use; line thickness corresponds to association strength. Core acupoints include Zhongji (CV3), Guanyuan (CV4), and Sanyinjiao (SP6). CV = conception vessel, SP = spleen meridian

Based on the above frequency analysis, the support degree was set to 2, and the confidence degree was ≥0.6 to conduct network topology analysis. Figure 4 shows a demonstration of the acupoint combination network. The results demonstrated that the Zhongji (CV3), Guanyuan (CV4), Sanyinjiao (SP6), Shenshu (BL23), and Qihai (CV6) acupoints constituted the core set of acupoints, characterized by their high frequency of combined utilization.

### 3.9. Clustering analysis of acupoints

**Figure 5.**
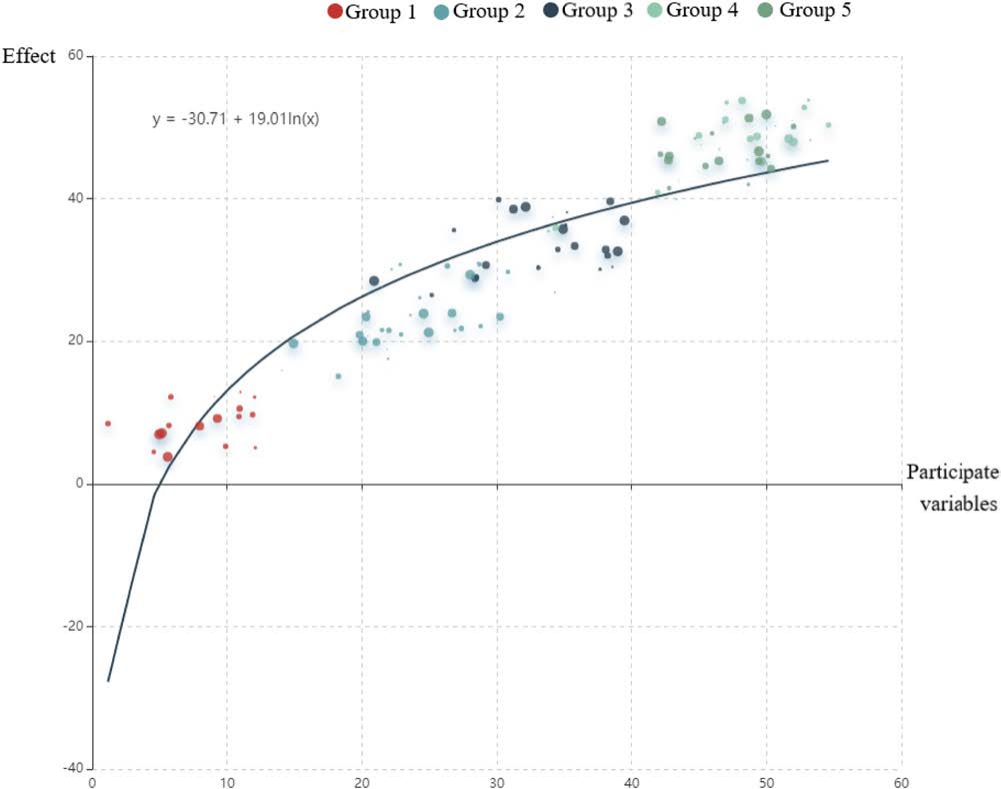
Clustering analysis of acupoints (K-means algorithm + cluster). 5 core clusters are color-coded (Group 1: red; Group 2: blue; Group 3: green; Group 4: purple; Group 5: orange). Cluster proximity to the regression curve (black line) indicates centrality of acupoints. Group 1 (red) aligns closely with core therapeutic combinations.

**Figure 6.**
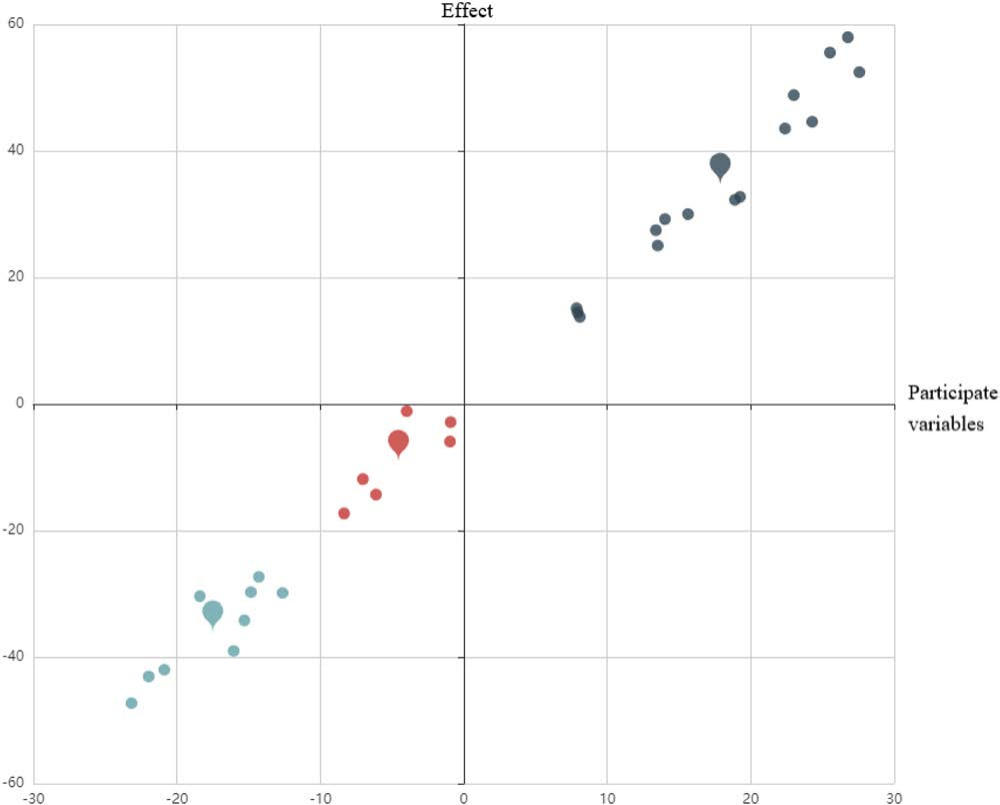
Clustering analysis of acupoints (K-means algorithm + Simulation of regression). Scatter plot with regression lines and confidence bands. Groups 2, 3, and 4 show dispersed distributions, while Group 5 (orange) contains the most prescriptions. Symbols (circles, triangles) denote cluster membership.

TCMISS was used for cluster analysis of the selected 85 articles. The number of clusters was set to 5, and 5 core groups were obtained. Core group 1 comprised Ciliao (BL32), Zhongliao (BL33), Huiyang (BL35), Shenshu (BL23), Pangguangshu (BL28), and Taichong (LR3). *Damp-heat accumulation* (urgency, dysuria). BL32/BL33 clears dampness via sacral modulation; LR3 drains liver fire. It can tonify the kidney qi, move the bladder meridian qi, activate blood, induce diuresis, and treat stranguria. Core group 2 included Guanyuan (CV4), Zhongji (CV3), Sanyinjiao (SP6), Shenshu (BL23), Yinlingquan (SP9), and Qihai (CV6). Spleen-kidney deficiency (frequency, nocturia). Integrates CV4/CV3 (tonify kidney Yang) + SP6 (fortify spleen) + BL23 (tonify kidney).^[29]^ It can deplete the kidney and fortify the spleen, regulate water passage, clear heat, and drain dampness, a commonly used combination for treating BPH. Reflecting the TCM treatment wisdom of “taking CV and governor’s vessel as the pivot, regulating 3 Yin to benefit water, and balancing Yin and Yang,” a trinity regulatory network of “innate-nurture (kidney)-nurture (spleen)-waterway (bladder)” was constructed. Core group 3 included Zhongji (CV3), Sanyinjiao (SP6), Guanyuan (CV4), Taixi (KI3), Zhibian (BL54), and Taichong (LR3). It mainly focuses on moving the bladder qi, tonifying the kidney qi, and promoting a lower-energizer, which embodies the integrated treatment concept of “tonifying the kidney and soothing the liver-promoting the regulation of the water channel – eliminating Yin and Yang” in the treatment of BPH. Core combination 4 included Guanyuan (CV4), Zhongji (CV3), Qihai (CV6), Qugu (CV2), Shuidao (ST28), and Shenshu (BL23). tonify the kidney and replenish qi, move qi, and drain dampness, reflecting the integrative treatment paradigm of TCM for BPH, namely “CV and governor vessel as key points – gasification sanjiao – Shu and mu Tongtiao.” The core group 5 was Dahe (KI12), Guanyuan (CV4), Taixi (KI3), Qihai (CV6), and Sanyinjiao (SP6), reflecting the integrated treatment concept of “tonify the kidney and Peiyuan – Xie Li Yin Yang – Tongtiao Chongren” in the treatment of BPH. Kidney essence depletion (advanced BPH with atrophy). KI12/KI3 nourish essence; CV4/CV6 consolidate chong-ren vessels. Furthermore, the k-means algorithm and regression model/clustering were combined to demonstrate the effect of clustering, as shown in Figures 5 and 6. Group 1 was close to the regression curve, Groups 2, 3, and 4 were more scattered, and Group 5 had more colors. This indicated that Group 1 was close to the core acupoints of this category, and Group 5 had more prescriptions.

### 3.10. Heterogeneity analysis and limitations

The current study acknowledges significant heterogeneity in acupuncture interventions, which may compromise the stability of pooled results. Primary sources of heterogeneity include: Technical variations, notably substantial disparities in electroacupuncture parameters (needling depth, retention time, and treatment course duration); intervention diversity, with 68% of studies incorporating adjunctive therapies (e.g., herbal medicine or tuina); and Inconsistent diagnostic criteria, evidenced by variable IPSS thresholds (8–20 points) for defining BPH severity. These methodological divergences may introduce bias in meta-analyses. Consequently, future trials should standardize intervention protocols – including needling depth and stimulation intensity – while adopting uniform outcome metrics (e.g., IPSS and QOL scores) to enhance evidence consistency.

## 4. Conclusion

In this review, TCMISS was used to conduct data mining on the literature related to acupuncture for BPH and to study the frequency of acupoint selection, frequency of meridian vessels, distribution rules of body parts, regulations of acupoint selection at specific points, association analysis, and cluster analysis of acupoints. It was concluded that the treatment of BPH: The commonly used acupoints for BPH are Guanyuan (CV4), Zhongji (CV3), Sanyiinjiao (SP6), Qihai (CV6), Shenshu (BL23), Shuidao (ST28), Pangguangshu (BL28), Yinlingquan (SP9), Qugu (CV2), Ciliao (BL32). The most commonly used meridians were the CV (173), bladder meridian (BL) (120), and spleen meridian (SP55). The most commonly used acupoints were the chest and abdomen (EX-CA) and back and waist (EX-BW). The most frequently used acupoints for the treatment of BPH were Guanyuan (CV4), Zhongji (CV3), Sanyinjiao (SP6), and Qugu (CV2). The most frequently used combinations are Zhongji (CV3) and Sanyinjiao (SP6). Guanyuan (CV4), Qihai (CV6); “Guanyuan (CV4), Sanyinjiao (SP6).” 5 core combinations were obtained: core combination 1: Ciliao (BL32), Zhongliao (BL33), Huiyang (BL35), Shenshu (BL23), Pangguangshu (BL28), Taichong (LR3); core group 2: Guanyuan (CV4), Zhongji (CV3), Sanyinjiao (SP6), Shenshu (BL23), Yinlingquan (SP9), and Qihai (CV6). Core Group 3 included Zhongji (CV3), Sanyinjiao (SP6), Guanyuan (CV4), Taixi (KI3), Zhibian (BL54), and Taichong (LR3). Core Group 4 included Guanyuan (CV4), Zhongji (CV3), Qihai (CV6), Qugu (CV2), Shuidao (ST28), and Shenshu (BL23). Core group 5 includes dahe (KI12), Guanyuan (CV4), Taixi (KI3), Qihai (CV6), and Sanyinjiao (SP6).

This study showed that acupuncture showed clinical efficacy for the treatment of BPH. The purpose of this study was to provide a basis and reference for acupoint selection in the treatment of BPH using acupuncture and to provide valuable information for further research and clinical applications. It also provides a reference for acupoint selection and prescription analysis of data-mining technology in the treatment of other diseases. This study elucidates novel therapeutic mechanisms of acupuncture.

## Acknowledgments

The authors are grateful to all participants in the present study.

## Author contributions

**Conceptualization:** Chen Guo, Jingwen Huang.

**Investigation:** Lifeng An.

**Methodology:** Geling Lu.

**Writing – original draft:** Chen Guo.

**Writing – review & editing:** Jingwen Huang.
